# Metal doped polyaniline as neuromorphic circuit elements for in-materia computing

**DOI:** 10.1080/14686996.2023.2178815

**Published:** 2023-02-27

**Authors:** R. Higuchi, S. Lilak, H. O. Sillin, T. Tsuruoka, M. Kunitake, T. Nakayama, J. K. Gimzewski, A. Z. Stieg

**Affiliations:** a International Center for Materials Nanoarchitectonics (WPI-MANA), National Institute for Materials Science (NIMS), Tsukuba, Japan; b Department of Chemistry and Biochemistry, University of California Los Angeles, Los Angeles, CA, USA; c Graduate School of Science and Technology, Kumamoto University, Kumamoto, Japan; d California NanoSystems Institute (CNSI), University of California Los Angeles, Los Angeles, CA, USA

**Keywords:** Nanoarchitectonics, neuromorphic, atomic switch, memristor, computing

## Abstract

Polyaniline-based atomic switches are material building blocks whose nanoscale structure and resultant neuromorphic character provide a new physical substrate for the development next-generation, nanoarchitectonic-enabled computing systems. Metal ion-doped devices consisting of a Ag/metal ion doped polyaniline/Pt sandwich structure were fabricated using an *in situ* wet process. The devices exhibited repeatable resistive switching between high (ON) and low (OFF) conductance states in both Ag^+^ and Cu^2+^ ion-doped devices. The threshold voltage for switching was>0.8 V and average ON/OFF conductance ratios (30 cycles for 3 samples) were 13 and 16 for Ag^+^ and Cu^2+^ devices, respectively. The ON state duration was determined by the decay to an OFF state after pulsed voltages of differing amplitude and frequency. The switching behaviour is analagous to short-term (STM) and long-term (LTM) memories of biological synapses. Memristive behaviour and evidence of quantized conductance were also observed and interpreted in terms of metal filament formation bridging the metal doped polymer layer. The successful realization of these properties within physical material systems indicate polyaniline frameworks as suitable neuromorphic substrates for *in materia* computing.

## GRAPHICAL ABSTRACT

**Figure:**
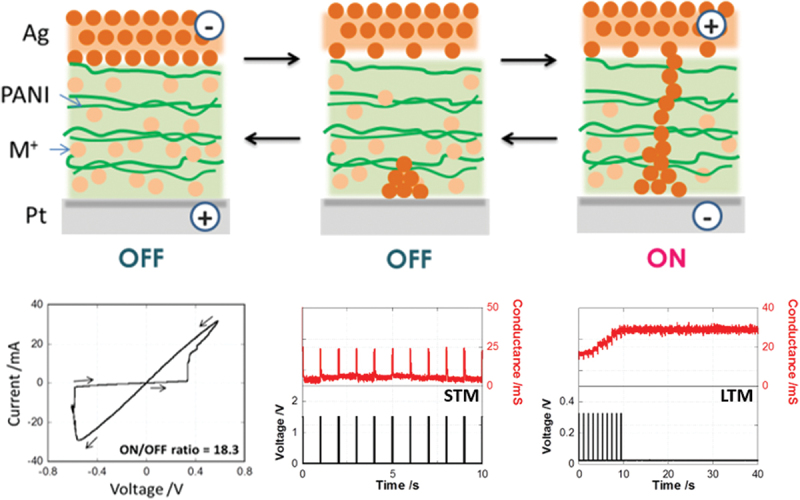

## 1. Introduction

In-materia computing is a burgeoning field of interest [1,2] that leverages the intrinsic, nonlinear dynamics of physical systems to perform complex computational tasks, with utility toward applications in reservoir computing (RC). RC is an adaptation of recurrent neural networks (RNN) which dynamically transform simple inputs fed through a reservoir into higher dimensional outputs alleviating the computational burden of conventional RNN training costs by only adjusting weights at the output layer. While RC has been traditionally implemented algorithmically, such software-based techniques are increasingly energy intensive and often require access to high-performance computing resources [3,4]. In contrast, hardware-based reservoirs offer a promising alternative by enabling the hardware itself to perform complex tasks in parallel, thereby alleviating bus latencies and lowering the computational overhead. Tailored material design using nanoarchitectonic principles can, in principle, be used to produce hardware-based reservoirs comprising functional nonlinear building blocks capable of performing computationally difficult tasks including speech recognition, waveform regression, and natural language processing [4–7].

Numerous material classes have been explored as candidate building blocks for in-materia RC. Electrochemical metallization cells (ECM) consisting of metal chalcogenides and valence change memory (VCM) using transition metal oxides have been explored [8,9], where recent progress has been made in addressing long-term stability challenges stemming from the controlled incorporation of oxygen in the latter class of materials [10]. Certain transition metal chalcogenide films utilize a tunable phase change to enable processing of both thermal and electrical energy as input signals [11]. Integration of various organic materials into devices has demonstrated useful properties including resistive switching in egg albumen [12] and threshold switching in silk-based biomaterials [13]. Polymer-based systems [14] including fluoropolymers [15], polyethylene oxide (PEO) [16–18] polyethylene dioxythiophene:polystyrene sulfonic acid [19,20] and polyaniline (PANI) [21,22] offer the opportunity to tune material properties, and thus resultant reservoir dynamics, by introducing different dopants and/or varying the optimum dopant levels and polymer chain length.

An ideal building block of devices for *in materia* RC should reliably exhibit (1) resistive switching between tunable states with high ON/OFF ratios as well (2) memory capabilities, including ‘synapse-like’ short- (STM) and long-term memory (LTM) [23–27]. Memory of previous inputs is particularly important for the implementation into physical RC frameworks that process temporal information based on their short- and long-term signal dependencies [28,29]. Various dielectric materials have demonstrated memristive switching when employing electrochemically active copper- and silver-based electrodes, where the selected dielectric influences the resultant temporal dynamics of the system as a direct consequence of the electrochemical and diffusive dynamics in the chemically active electrode materials [30]. Tailoring the dielectric medium thereby offers the opportunity to optimize device dynamics toward task-specific computation. Organic, conducting polymers are promising candidate materials that offer a large degree of tunability based on their chemical properties, particularly when use in combination with electrochemically active electrode materials [31].

**Figure 1.**
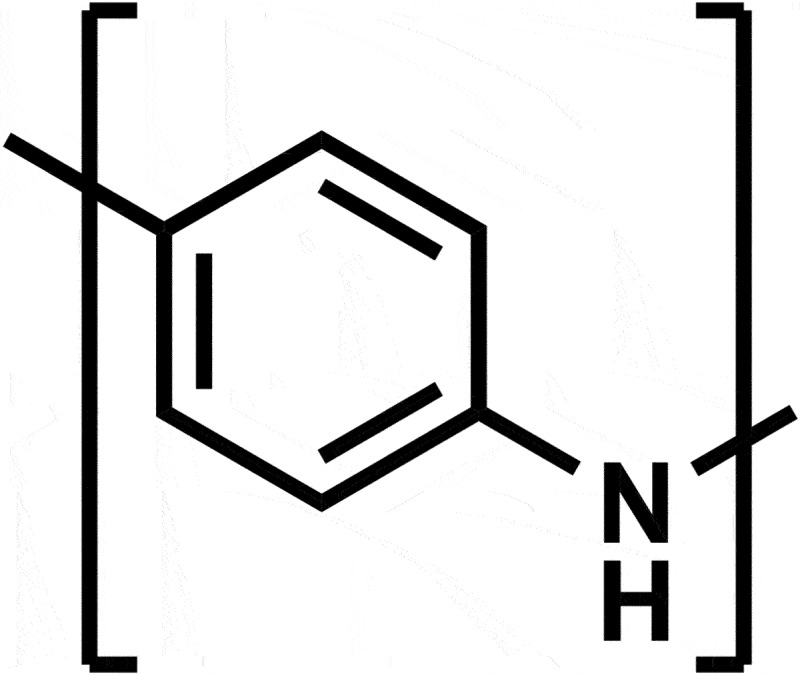
Chemical structure of polyaniline.

Polyaniline (PANI), whose chemical structure is shown in Figure 1, is an intrinsically conducting polymer that offers superior chemical and thermal stability in addition to solubility in aqueous media [32–37]. The extensive use of PANI in a broad range of industrial applications, including printed circuit board manufacturing, anti-corrosion agents and antistatic coatings, has produced an expansive database of its thermal, electrical and material properties as well as long-term stability under a wide range of conditions. PANI-based sensors have also demonstrated compatibility with complementary metal oxide semiconductor (CMOS) technology, suggesting solution-based processing of PANI to be compatible with traditional CMOS processing [38,39]. Synthetic control of PANI morphology has been shown to produce particle, film, and fiber-based materials as well as the opportunity to tune electronic properties through optimization of polymer chain length [40–42]. PANI has also garnered interest as a memristive circuit element [43] and has been shown to exhibit spike-timing dependent plasticity (STDP) [44–46]. Consequently, PANI has recently been used as the material building block for a basic *perceptron* [47].

Herein, we present the operational properties (electrical and memory) of polyaniline-based atomic switches designed using nanoarchitectonic concepts and prepared by a simple, *in situ* process that incorporates metal (silver or copper) cationic dopants. The resulting metal doped PANI devices exhibited reproducible, characteristic memristive switching between high (ON) and low (OFF) conductance states. The memory capacity of these materials was monitored as a function of time, where applied bias voltages successfully modulated the duration of the ON state to demonstrate both STM and LTM. These unique PANI-based devices also exhibited conductance quantization. Collectively, these operational characteristics implicate metal doped PANI thin films as a suitable building block for the development of new physical substrates for *in materia* computing.

## 2. Experimental

### 2.1 Materials

Aniline and copper chloride were purchased from Sigma Aldrich, and silver nitrate was purchased from Fisher Scientific. These reagents were used without further purification.

### 2.2 Preparation of metal ion-doped PANI films

PANI layers were prepared by *in situ* polymerization on platinum patterned on the surface of a silicon wafer. The substrate was rinsed with acetone and ultra-pure water, and then dried in air. In the case of the Ag-doped PANI [48], 5 mL of 0.2 M aniline and 0.02 M silver nitrate aqueous solution was used. For Cu-doped PANI [49], 5 mL of 0.1 M aniline and 0.01 M copper chloride aqueous solution was used, which are final concentrations. The substrate was immersed in the solution for 24 hours at 30°C without stirring soon after preparing the solution. The substrate was removed from solution and rinsed in ultra-pure water and then dried in a desiccator. The thickness of the PANI-Ag^+^ and PANI-Cu^2+^ layers were 160 nm and 80 nm, respectively.

### 2.3 Device integration and electrical characterization

Colloidal silver paste (PELCO Colloidal Silver, TED PELLA) applied to the metal sulfide layer or metal ion-doped PANI layer and served as a top electrode. The electrode area of silver layer was~1 mm^2^. The device was then dried in desiccator overnight. Top electrode and bottom electrodes were contacted using probe electrodes whose position was precisely controlled using a micro-manipulator. Current – voltage (*I – V* spectroscopy) was recorded using a National Instruments 4132 unit, while time series *I-V* data was collected using an analog voltage input/output module (National Instruments 6368) in conjunction with a current-to-voltage preamplifier (Stanford Research Systems SR570). A part of pulse measurements was carried out using a sub-femtoamp remote source meter (Keithley 6430). ON/OFF ratios were determined using the ON and OFF state conductance, respectively, calculated using a least-squares fitting of the corresponding region in the I-V curve.

## 3. Results and discussion

Two different metal dopants were employed to assess the suitability of PANI as an *in materia* computing substrate, Type-I devices comprising diffused Cu^2+^ ions and Type-II devices comprising Ag^+^ ions. These metal ion-diffused devices comprise a PANI matrix layer in which metal ions are embedded by physical adsorption and/or the formation of coordination bonds with the PANI backbone. Polymer layers prepared using *in situ* polymerization from an aqueous solution of aniline containing the metal salt without acid formed metal ion-diffused PANI layers as confirmed by UV-Vis adsorption spectra [50] and scanning electron microscopy (SEM) as shown in Figure S1.

### 3.1 Type I devices

**Figure 2.**
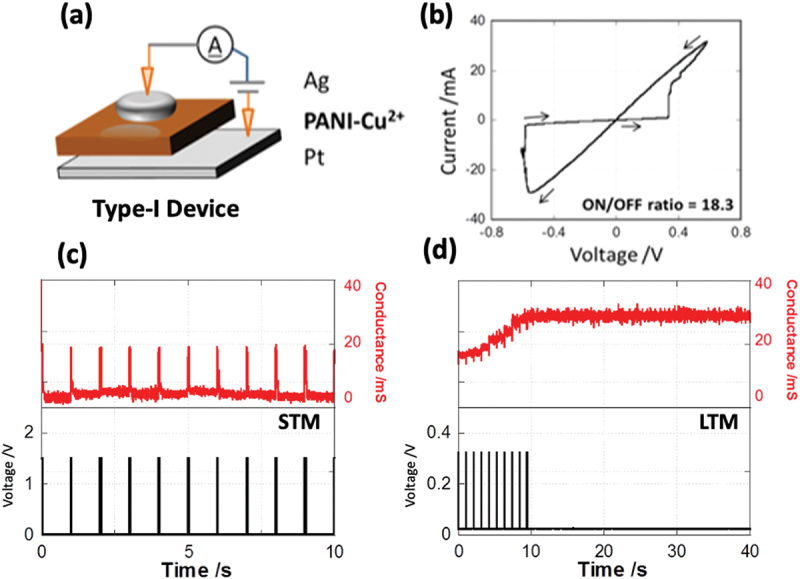
(a) Device architecture for Type I: Cu^2+^- doped devices; (b) *I-V* response to ± 0.6 V input at 1 hz sweep rate; (c, d) Conductance response of pulsed inputs of width (W) = 0.05 s at intervals (T) = 1 s for voltage amplitudes (V) = 1.5 V (c, STM) or 0.3 V (d, LTM).

A representative *I-V* curve for the Type-I Ag/PANI-Cu^2+^/Pt device architecture (see Figure 2(a)) shown in Figure 2(b) demonstrates switching between high conductance (ON) and low conductance (OFF) states. The voltages required to switch between the ON and OFF states, the SET voltage (V_SET_), were 0.34 and −0.56 V, respectively. An average ON/OFF conductance ratio for Type-I devices of 16.0 was observed across 30 samples within the calculated standard deviation. Use of *in situ* polymerization process allows for control of film composition, morphology, and thickness by simple tuning of the synthetic conditions. For example, the initial concentration of metal ions used during film formation was found to affect the switching characteristics. For example, a ratio of ~ 1500 for ON/OFF states was achieved for Type-I by changing the initial concentration of the aniline precursor and metal dopants (Figure S2).

To explore memory capacity in Type I devices, a sequence of voltage pulses was utilized to discern the duration of higher conductance states. By varying the inter-pulse interval while maintaining a constant amplitude of voltage and pulse length, device conductance temporarily increased and subsequently relaxed to its initial state after termination of the voltage input (Figure 2(c)). The ON state persisted for less than 0.5s, and the application of multiple pulses did not create a persistent ON state. This volatile switching behaviour represents what has been referred to as STM in atomic switches and other memristive devices [51]. Conversely the application of voltage pulses with a shorter inter-pulse interval induced a constant, high-conductance state that persisted for more than 30s (Figure 2(d)). These behaviours are attributed to previously observed LTM. These results demonstrate that PANI-Cu^2+^ layers exhibit synaptic-like neuromorphic behaviour, where the input frequency controls the two states of memory retention.

### 3.2 Type II devices

**Figure 3.**
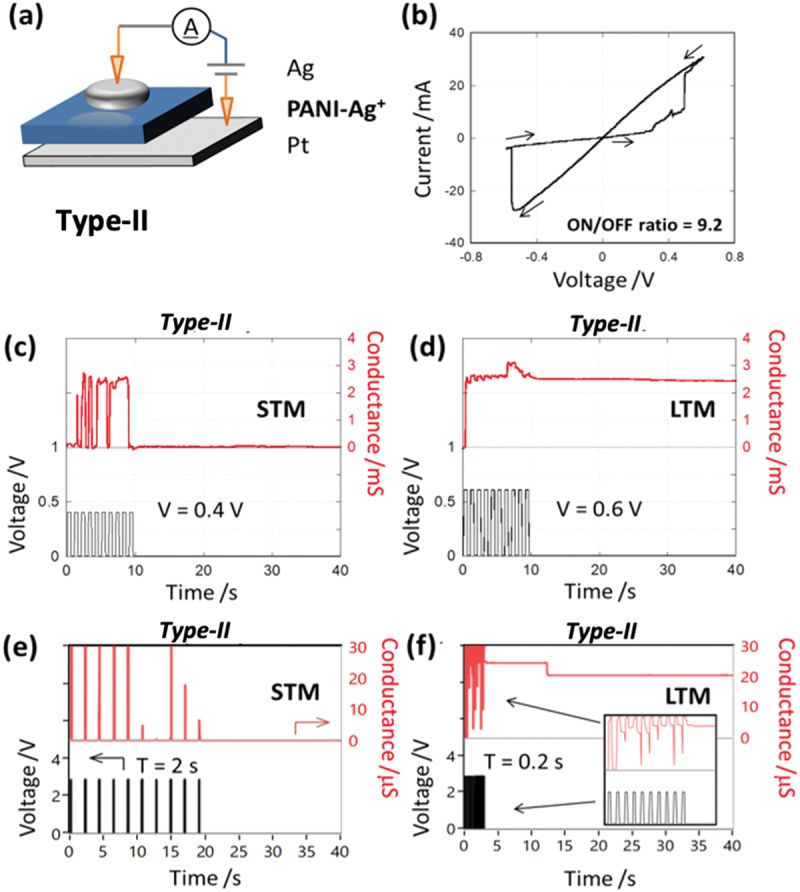
(a) Device architecture for Type II: Ag^+^- doped devices; (b) *I-V* response to ± 0.6 V input at 1 hz sweep rate.; (c – f) conductance response pulsed input. (c) input voltage (V) = 0.4 V, pulse width (W) = 0.5 s, interval (T) = 0.5 s; (d) V = 0.6 V, W = 0.5 s, *T* = 0.5 s; (e) V = 2.9 V, W = 0.1 s, *T* = 2 s; (f) V = 2.9 V, W = 0.1 s, *T* = 0.2 s.

A representative current-voltage (*I-V*) curve for a Type-II device, consisting of a Ag/PANI-Ag^+^/Pt architecture (see Figure 3(a)), shown in Figure 3(b) reveals SET voltages (V_SET_) of 0.50 V and −0.56 V for the ON and OFF states, respectively. An average ON/OFF conductance ratio for Type-II devices of 13 was observed across thirty voltage sweeps, with a concurrent decrease in the SET voltage after repeated switching. This behaviour is attributed to the initial formation of a metallic filament under an applied positive bias followed by dissolution of the deposited metal atoms under negative bias, where a small fraction of the filament remains within the timescale of the applied 1 Hz sweeps used. Accordingly, the SET voltage decreased by the 2nd sweep as has been reported for other classes of polymeric atomic switches [18].

To explore memory capacity in Type II devices, voltage pulse sequences were again utilized to discern the duration of higher conductance states, which was found to depend on both the amplitude and frequency of the input signal (Figure 3(c-f)). Two distinct memory tests were carried out using pulsed input voltages: (1) the voltage amplitude was varied while maintaining a constant pulse and inter-pulse time interval length of 0.5 s, and (2) the inter-pulse time interval was varied while maintaining a constant amplitude of voltage and pulse length. In the former case, STM was observed (Figure 3(c)) whereby device conductance temporarily increased and subsequently relaxed to its initial state after termination of the voltage input (0.4 V). The ON state persisted for less than 0.5s, and the application of multiple pulses did not create a persistent ON state as revealed in the magnified profile (Figure S3). Conversely, the application of larger amplitude voltage pulses (0.6 V) induced LTM (Figure 3(d)), denoted by a constant, high-conductance state that persisted for more than 30s, where the conductance decreased slightly with each interval between pulses (Figure S4).

The STM/LTM behaviour was also found to be determined by inter-pulse time intervals. Specifically, low-frequency pulses induced STM (Figure 3(e)) while high-frequency pulses induced LTM (Figure 3(f)). These results demonstrate that PANI-Ag^+^ layers also exhibit synaptic-like behaviour where both the input voltage amplitude and frequency control the two states of memory retention. We propose that the pulses of higher amplitude or frequency lead to the formation of numerous and/or thicker metallic nano-filaments in the PANI-Ag^+^ layer bridging the electrodes [52].

**Figure 4.**
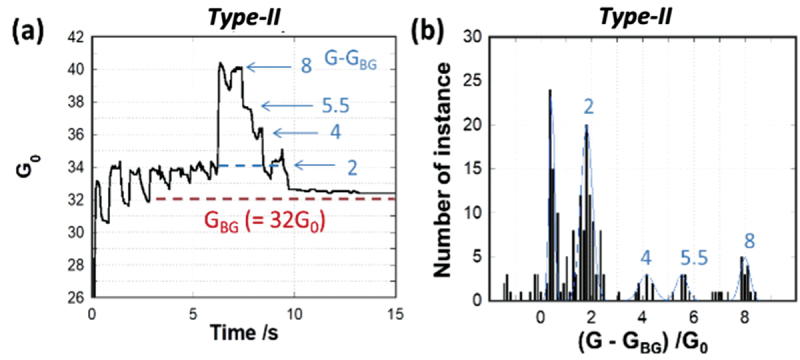
(a) Magnified region of the pulse response for Type-II devices (Figure 1d). (b) Histogram of G_0_ fitted by Gaussian curve (blue line). The number corresponds to each step observed in the change of conductance.

Detailed observation of the initial period (0–15s) in conductance-time plots for Type-II (Figure 3(d)) revealed distinct plateau regions and steps. A magnified plot is shown in Figure 4(a), where conductance has been converted into units of G_0_ based on the point contact conductance G_0_ (G_0_ = 2*e* [2]/*h* = 77.5 µS, where *e* is the fundamental electron charge and *h* is Planck’s constant) [53]. Here, the leakage current induced by ion conduction or short-circuiting was included as an additional resistor in parallel. The leakage current was calculated to be 20 kΩ based on the measured resistance in the OFF state of the device. A conductance histogram of G_0,_ plotted using data from Figure 3(a), is shown in Figure 3(b), where conductance was recalculated by subtracting this background conductance. Four peaks at 2.8, 4.1, 5.5, and 7.9, fitted to Gaussian curves, were assigned to each increment observed in G_0_ as shown in Figure 4(a). The peak-to-peak differences corresponded to integer multiple of G_0_ (3, 4 and 8) except for peak at 5.5. These discrete changes in conductance indicate the presence of quantized conductance steps.

To confirm the robustness of these conductance jumps, repeated measurements under equivalent conditions were similarly considered and plotted as a conductance histogram (Figure S5). Integer multiples of G_0_ were again observed in the peaks of the conductance histogram, indicating that that device conductance is quantized by stepwise formation and growth of Ag filaments across the PANI-Ag^+^ layer in a similar fashion to conductance quantization previously reported in Ta_2_O_5_-based [54], PEO-based atomic switches [55] and Cu/SiO_2_/W memristor devices [56]. While it is quite difficult to observe the size or shape of filaments experimentally [16,57], Krishnan *et al*. have reported that the 9-atom chain bridging electrodes can lead 5 G_0_ − 9 G_0_ based on the simulation for a polymer-based atomic switch [55]. According to this previously reported work, it is reasonable to attribute the discrete changes of 1 G_0_ − 8 G_0_ observed in Type-II devices (Figure 4(b)) to incremental filament growth.

### 3.3 Mechanistic validation

**Figure 5.**
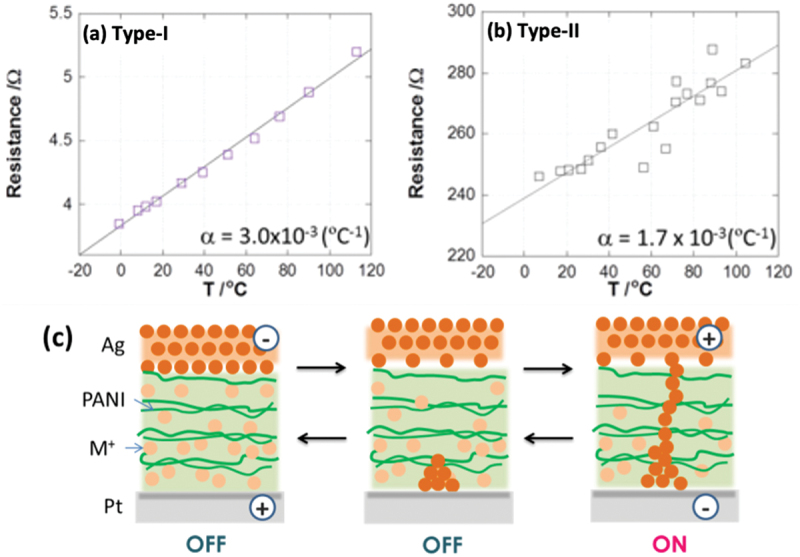
(a-b) Temperature dependence of resistance the on states. (c) Proposed mechanism of resistive switching for metal ion-doped device.

The filamentary switching properties of both Type-I and Type-II devices were further explored by monitoring changes in device resistance as a function of temperature. The typical temperature dependences of resistance in their ON state for Device-I and Device-II are shown in Figure 5(a-b), where resistances were calculated from the slopes of *I-V* curves in the ON state with varying temperature. For both devices, the resistance was observed to linearly increase with increasing sample temperature. These plots were then fitted to the equation for metallic resistivity, R(T) = R_0_ (1 + αT), where R_0_ is the resistance at 0°C, and α is the temperature coefficient of the resistivity. The α value of each device calculated from this equation was 3.0 × 10^−3^ for Type-I and 1.7 (±0.1) × 10^−3^ for Type-II devices. These values agree with reported values of 4.4 × 10^−3^ °C^−1^ for Cu and 4.2 × 10^−3^ for Ag coefficients [58].

The variation of resistance, in the ON state, at each switching event and the increase in resistance induced by grain boundaries in the metal filament may have a minor influence on the α coefficient. Nevertheless, the closeness of the results to the coefficients for bulk Ag or Cu and their linear correlation supports the assignment that the high conductance in the ON state is induced by metal filaments formed across the ion doped PANI layer. It is therefore expected that the metal ions embedded in the PANI layer are deposited as the metal filaments by electrochemical reduction to create the ON state when a voltage is applied (Figure 5(c)). In contrast, reverse polarity inputs result in electrochemical oxidation and dissolution of metal filaments, leading to the OFF state.

The phenomenon of Ag filament formation in a polymer matrix has been investigated by Krishnan *et al* [16]. PANI is expected to be in a emeraldine base state which is not conductive, so that PANI should be regarded as a passive insulating layer in a metal-insulator-metal structure. The similarity between Ag+- and Cu^2+^-doped devices indicates that the amounts and quality of metal filaments formed across the metal ion-doped PANI layer are similar regardless of the ion species. Importantly, doped PANI-Ag^+^ and PANI-Cu^2+^ show that embedded metal ions in polymer film, added during *in situ* polymerization, predominantly contributes to the formation of metal filaments and that the Ag top electrode does not serve as significant source of metal filaments for resistive switching in this system. The ON-OFF resistance ratios of both device types were sufficient to distinguish each state and can likely be improved by reducing ion conduction and/or leakage background currents in the OFF state.

## 4. Conclusions and outlook

PANI-based atomic switches exhibit reproducible, robust resistive switching and synapse-like memory characteristics including both short- (STM) and long-term (LTM) memory. These essential properties are known to enable the processing of temporal signals by the physical substrate for *in materia* computing applications. *In situ* processing of these metal-doped polymers provides a path toward controlled and area-selective fabrication of complex resistive switching nanoarchitectures on arbitrary scales. The successful incorporation of different metal dopants into operational device architectures implies a general versatility for using other ionic species and offers the opportunity to modulate the properties of PANI-based devices including memristive switching characteristics, ON/OFF ratios, and volatility. This class of metal-doped polymers provide a new, promising pathway for the fabrication of neuromorphic building blocks as substrates for *in materia* computing while maintaining compatibility with modern CMOS processing and hardware technologies.

## Supplementary Material

Supplemental Material

Supplemental Material

Supplemental Material

Supplemental Material

Supplemental Material

Supplemental Material
