# Supplementary figures and images for: Metal doped polyaniline as neuromorphic circuit elements for in-materia computing
Source: Sci Technol Adv Mater. 2023 Feb 27;24(1):2178815. doi: 10.1080/14686996.2023.2178815 (PMC9980013; doi:10.1080/14686996.2023.2178815)

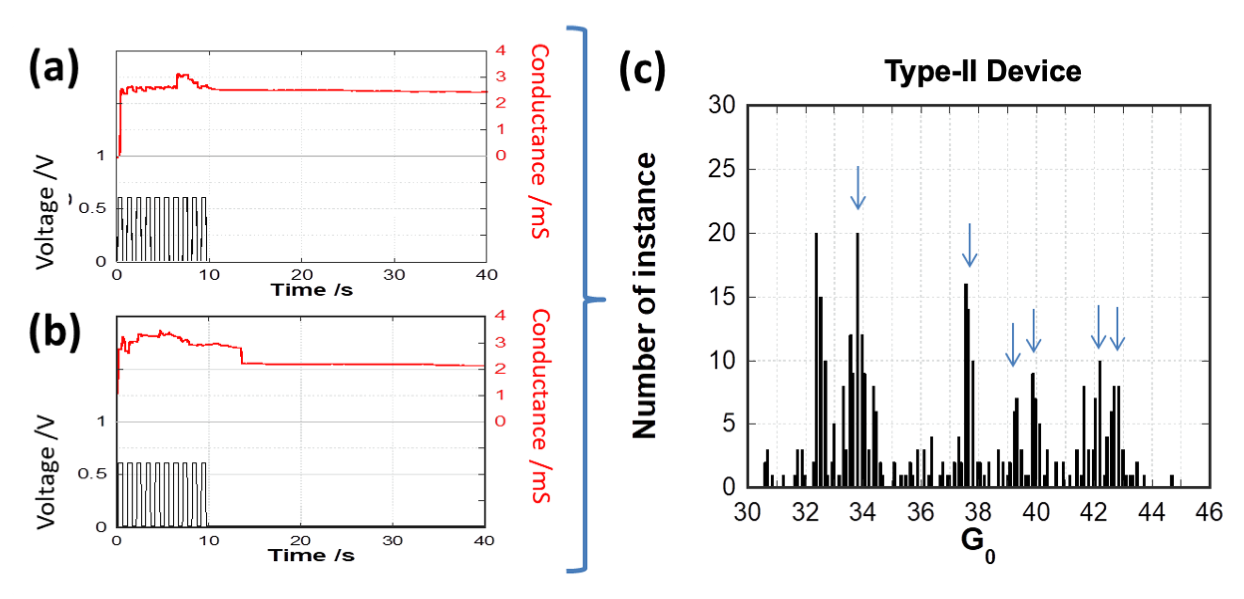

Supplement: Supplemental Material [file TSTA_A_2178815_SM3627.png]

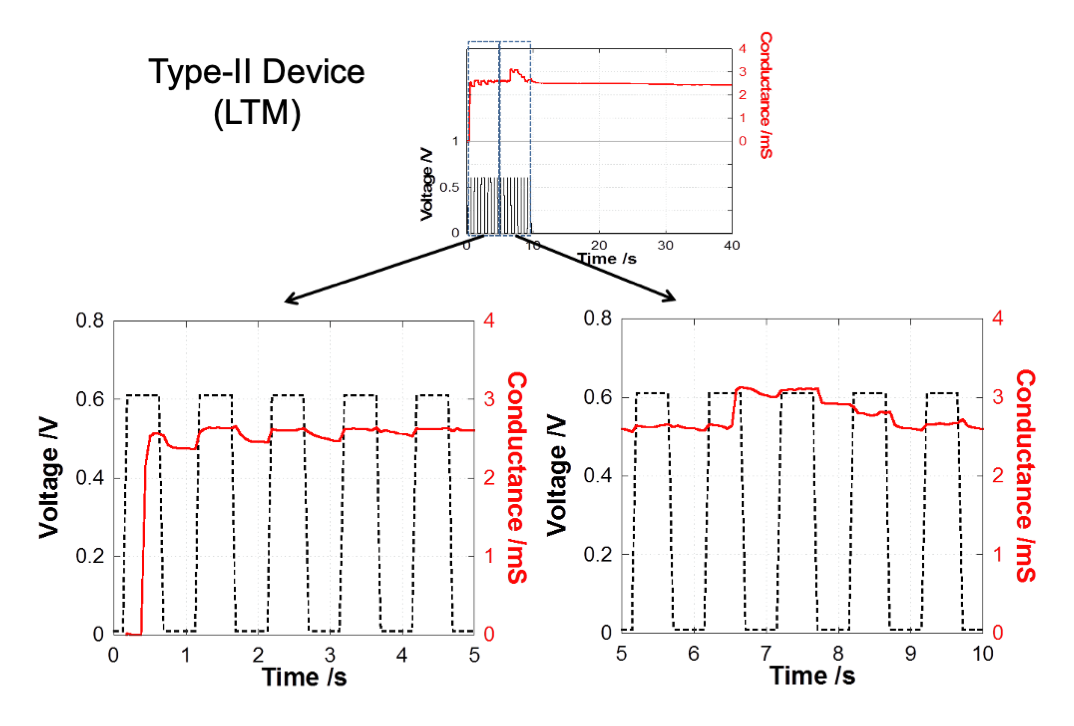

Supplement: Supplemental Material [file TSTA_A_2178815_SM3626.png]

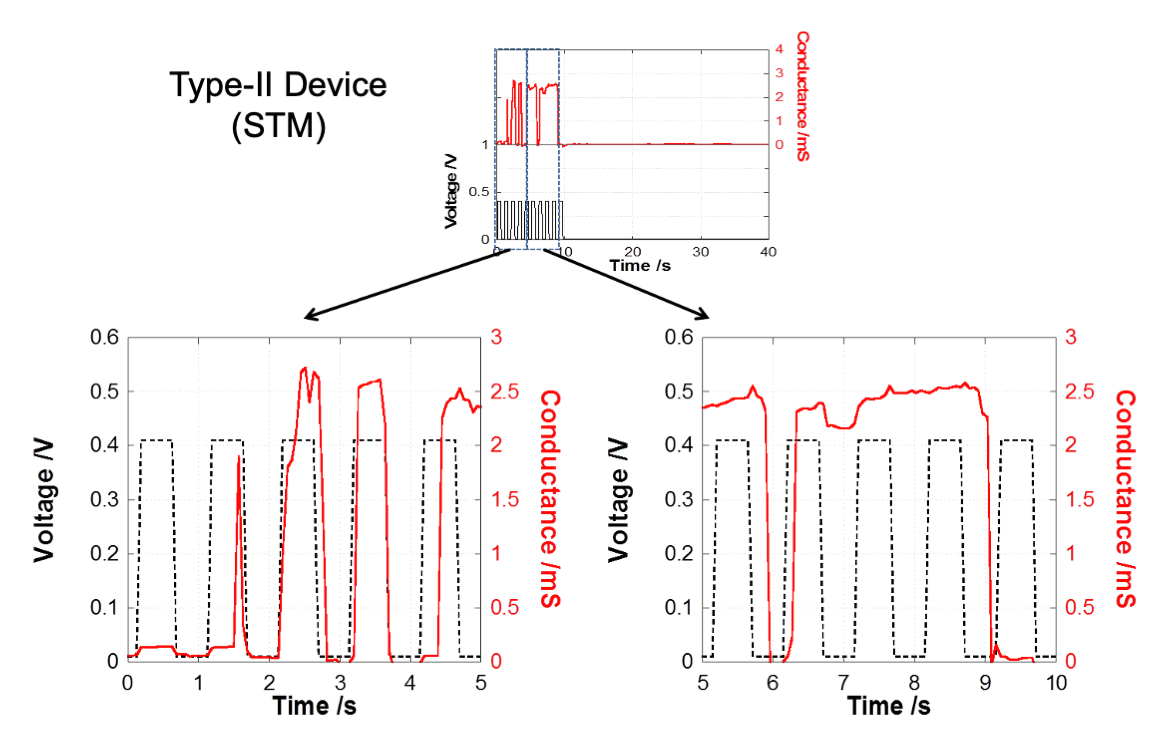

Supplement: Supplemental Material [file TSTA_A_2178815_SM3625.png]

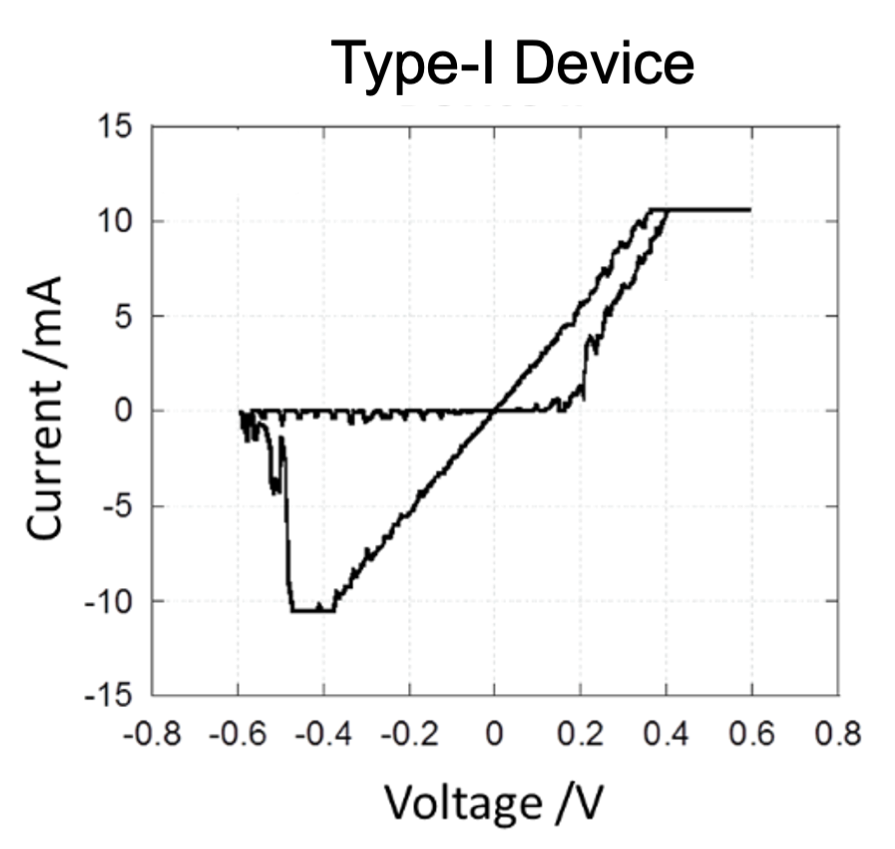

Supplement: Supplemental Material [file TSTA_A_2178815_SM3624.png]

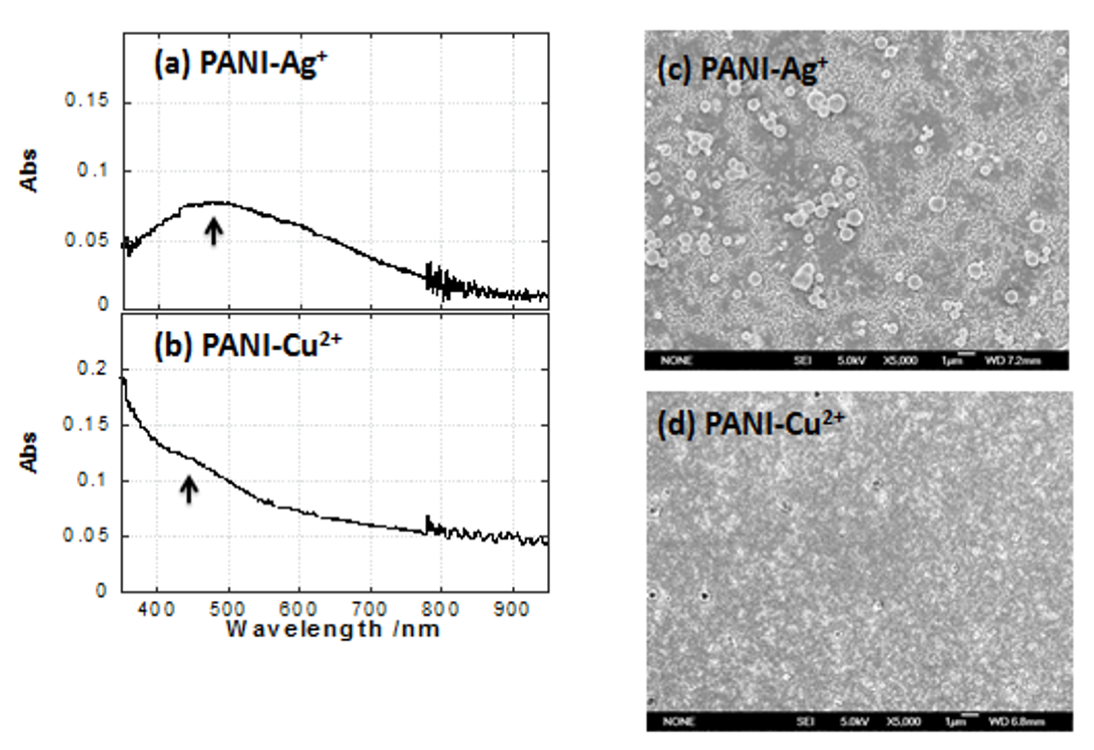

Supplement: Supplemental Material [file TSTA_A_2178815_SM3620.png]
