# Supplementary material for: Metal doped polyaniline as neuromorphic circuit elements for in-materia computing
Source: Sci Technol Adv Mater. 2023 Feb 27;24(1):2178815. doi: 10.1080/14686996.2023.2178815 (PMC9980013; doi:10.1080/14686996.2023.2178815)
Supplement: Supplemental Material [file TSTA_A_2178815_SM3616.docx]

**Supplementary Information**

*Metal-doped polyaniline as neuromorphic circuit elements for in materia computing*

R. Higuchi^a^, S. Lilak^b^, H. O. Sillin^b^, T. Tsuruoka^a^, M. Kunitake^c^, T. Nakayama^a,d^, J. K. Gimzewski*^a,b,e^ and A. Z. Stieg*^a,e^

*^a^ International Center for Materials Nanoarchitectonics (WPI-MANA), National Institute for Materials Science (NIMS), 1-1 Namiki, Tsukuba, 305-0044, Japan*

*^b^ Department of Chemistry and Biochemistry, University of California Los Angeles 607 Charles E. Young Drive East, Los Angeles, CA 90095, USA*

*^c^ Graduate School of Science and Technology, Kumamoto University, 2-39-1 Kurokami, Kumamoto 860-8555, Japan*

*^d^ Graduate School of Pure and Applied Sciences, University of Tsukuba, 1-1 Namiki, Tsukuba, Ibaraki 305-0044, Japan*

*^e^ California NanoSystems Institute (CNSI), University of California Los Angeles, 570 Westwood Plaza, Los Angeles, CA 90095, USA*

Figure S1. UV-Vis absorption spectra were measured to confirm the PANI-Ag^+^ and PANI-Cu^2+^ thin films successfully deposited by *in situ* process. The absorption peak at 470 and 440 nm can be assigned to adsorption by PANI-Ag^+^ and PANI-Cu^2+^ film, respectively.

SEM image shows inhomogeneous surface morphology which obviously different from bare substrate, glass slide; the results also support the growth of polymer films.


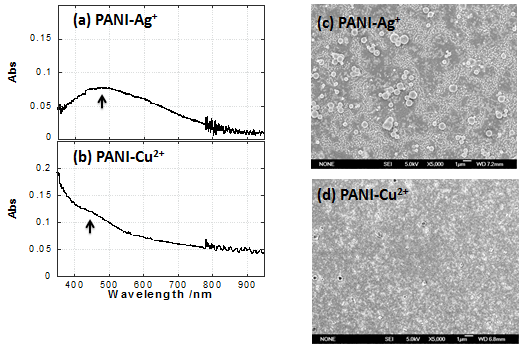


S1. UV-Vis absorption spectra of (a) PANI-Ag^+^ and (b) PANI-Cu^2+^ film deposited on the glass slide. The arrows point peaks. (c-d) SEM image of surface structure for PANI-Ag^+^ and PANI-Cu^2+^ film on Pt electrode.

Figure S2. The initial concentration of aniline and metal ion affected the ratio of ON/OFF resistance. In the preceding experiment (Fig. S1), resistance of the OFF state was increased by reducing the initial concentration by half, which dramatically increased the ON/OFF ratio.


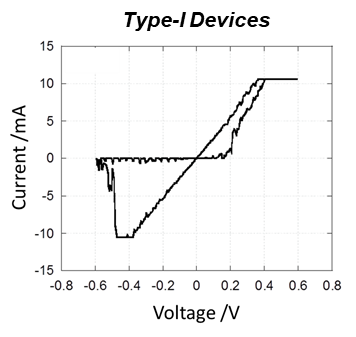


S2. *I*-*V* curve with higher ON/OFF ratio of ~1500 was obtained by sweeping a voltage of 0.6 V at 1 Hz for Type-I devices. The initial concentration of aniline and CuCl_2_ was optimized around 0.1 and 0.01 mM, respectively.

Figure S3 and S4. Magnified plots of the STM/LTM measurements show that the pulsed input certainly increased the conductance. In addition, the decay of conductance was observed at each interval between pulses.


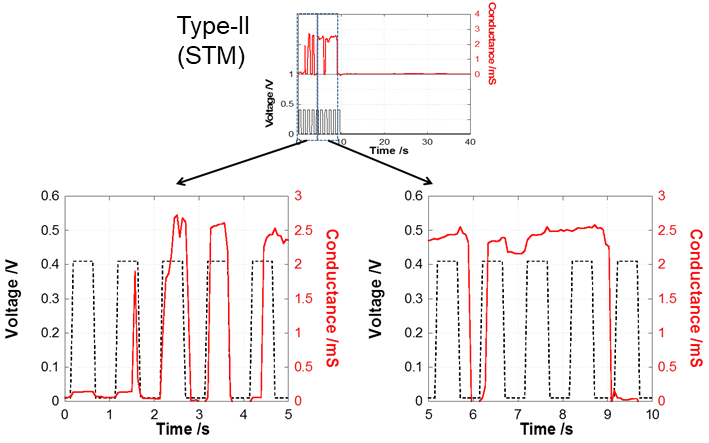


S3. Magnified plots from Figure 1c of applied voltage and conductance change observed in Type-II devices under pulsed bias stimulation. The dotted line (black) is voltage and solid line (red) is conductance.


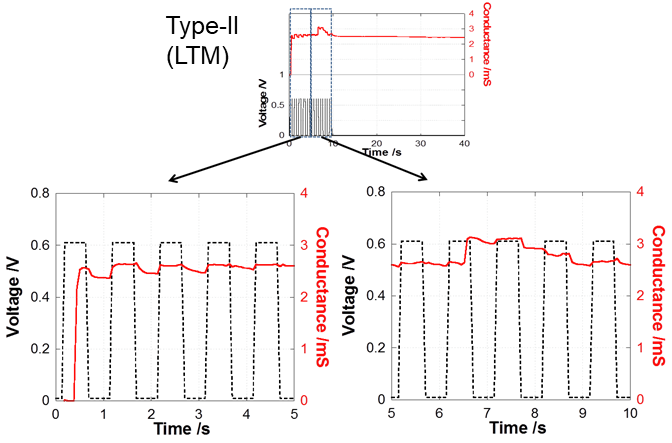


S4. Magnified plots from Figure 1d of applied voltage and conductance change observed in Type-II devices under pulsed bias stimulation. The dotted line (black) is voltage and solid line (red) is conductance.

Figure S5. Histogram of device conductance under pulsed bias stimulation (Fig. S4c) confirms the robustness of conductance quantization. The histogram shows several peaks at integer multiples of G_0_ (G_0_ = 2*e*^2^/*h* = 77.5 µS, where *e* is the fundamental electron charge and *h* is Planck’s constant). This result supports the conductance quantization in PANI-Ag^+^ device.


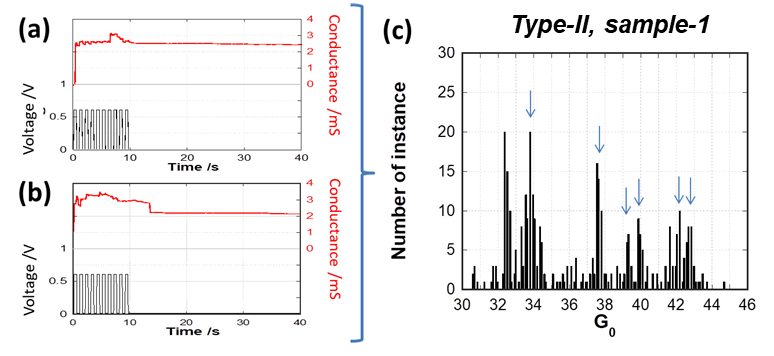


S5. (a, b) Conductance responses to input pulsed voltage on same sample. (c) Histogram of G_0_ obtained by two sets of conductance data from 0 to 13 s in both (a) and (b). The blue arrows indicate the discrete peaks. The input voltage condition is as follows; Voltage = 0.4 V, pulse width = 0.5 s, interval = 0.5 s.
